# NK cell dysfunction in antiphospholipid syndrome

**DOI:** 10.3389/fimmu.2025.1593705

**Published:** 2025-06-12

**Authors:** Anush Martirosyan, Eva Kriegova, Gayane Manukyan

**Affiliations:** ^1^ Laboratory of Molecular and Cellular Immunology, Institute of Molecular Biology, National Academy of Sciences, Yerevan, Armenia; ^2^ Department of Immunology, Faculty of Medicine and Dentistry, Palacký University Olomouc and University Hospital Olomouc, Olomouc, Czechia

**Keywords:** antiphospholipid syndrome, antiphospholipid antibodies, NK cells, placenta, peripheral blood

## Abstract

Antiphospholipid syndrome (APS) is a systemic autoimmune condition characterized by the persistent presence of antiphospholipid antibodies (aPL), and is commonly associated with thrombosis and pregnancy-related complications. To date, relatively little is known about the potential of NK cells in mediating the pathological effects of APS. While the role of NK cells in controlling immune responses and maintaining tissue homeostasis is relatively clear, the fact that they are also linked to various autoimmune conditions is now being highlighted. Given the impact of NK cells on immune regulation, vascular function, and pregnancy outcomes, the unifying message of a critical role for NK cells in APS emerges. As innate immune cells, NK cells might be activated in an antibody dependent manner and exert antibody-dependent cellular cytotoxicity (ADCC). In this process, NK cells recognize and bind to the Fc portion of antibodies that have attached to target cells. With their immunoregulatory properties in the uterus, NK cells play a crucial role in facilitating endometrial tissue remodeling, supporting vascular function, and contributing to placental formation, all of which are essential for a successful pregnancy. In APS, the presence of aPL may disrupt the delicate balance of NK cell-mediated immune regulation leading to alterations in cell activation, cytokine production, and cytotoxic functions. Given the multifactorial nature of NK cells in peripheral blood and uterus, the review provides insight into the potential underlying mechanisms through which NK cells may contribute to thrombosis and pregnancy complications in APS.

## 1 Functional plasticity of NK cell subsets across physiological and autoimmune conditions

In human peripheral blood, there are two major subgroups of NK cells based on their differential expression of CD16 and CD56. The predominant CD16+CD56^dim^ subset is primarily recognized for its cytotoxic functions, whereas the minor CD16−CD56bright is notable for its robust cytokine production, similar to CD4+ T helper cells. The former one is more abundant in secondary lymphoid tissues and in immunotolerant organs, such as liver, lung and uterus (1). Additionally, a distinct subset of NK cells that produces IL-10 is referred to as NK regulatory or adaptive cells. These cells possess immunosuppressive properties and are involved in immune-regulatory processes (2, 3). NK cell activation is determined by a delicate balance of activating and inhibitory receptors that regulate their cytotoxic function. Cytokine-producing NK cells express high levels of the inhibitory CD94/NKG2A complex that recognizes HLA-E, and undergo intensive proliferation in response to IL-2 or IL-15. Although they contain high numbers of cytolytic granules, they respond poorly to target cell stimulation at steady state. Cytotoxic NK cells express MHC class Ia allele-specific killer cell Ig-like receptors (KIRs) and display a strong cytolytic activity and cytokine secretion capability rapidly upon activation (4, 5). Contact-dependent cytotoxicity of NK cells occurs through release of cytolytic granules containing granzymes perforin, FasL, tumor necrosis factor (TNF)-related apoptosis-inducing ligand (TRAIL), and granulysin into target cells, leading to apoptosis through both caspase-dependent and caspase-independent mechanisms (6). NK cell activation occurs through three main mechanisms: i) antibody-dependent cellular cytotoxicity (ADCC) by binding to the Fc portion of IgG antibodies coating target cells; ii) recognition of altered molecules on stressed cells; iii) cytokine signaling in microenvironment abundant in cytokines like IL-12, IL-15 or type I interferons (7).

**Figure 1:**
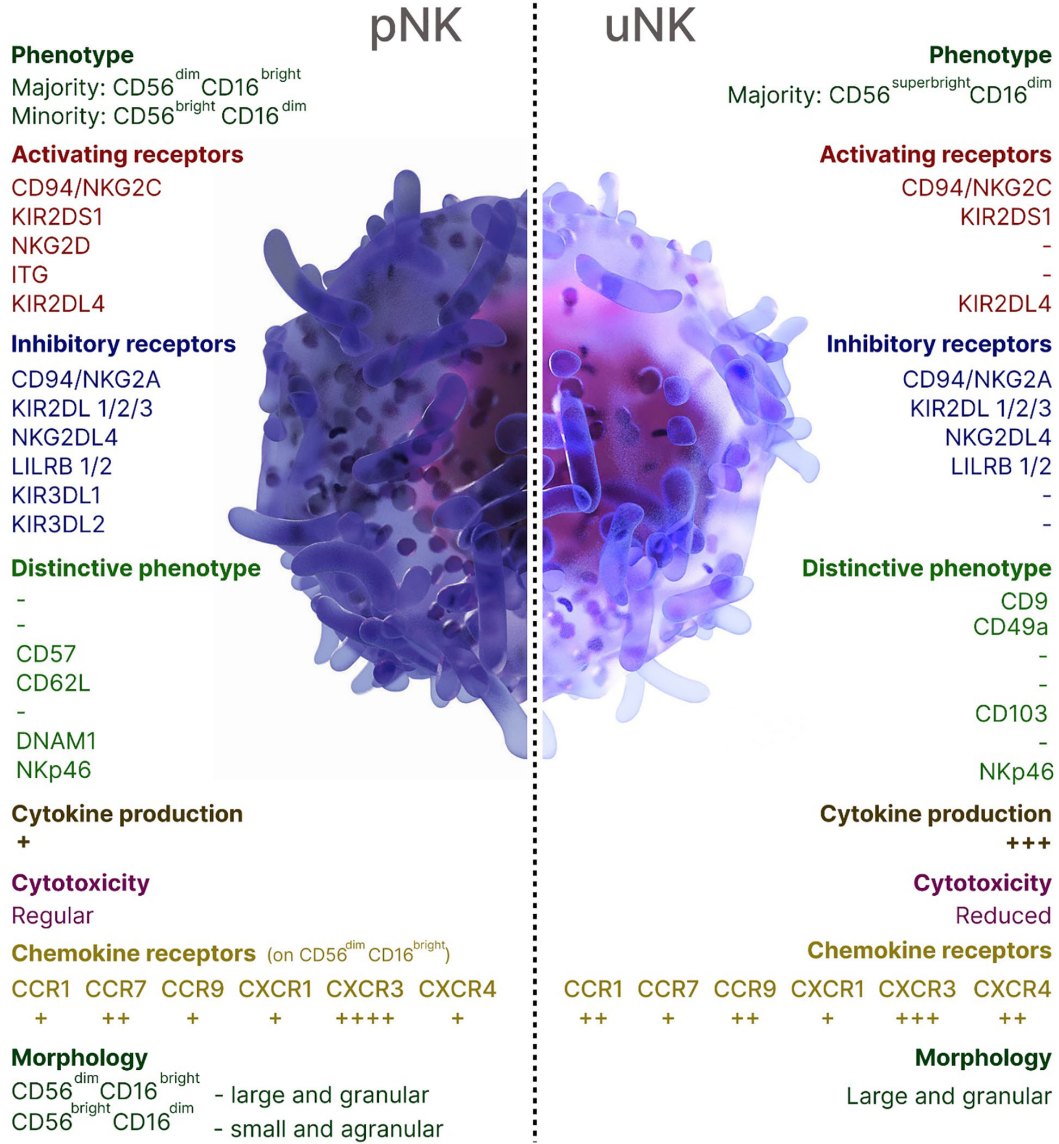
Overview of peripheral blood and uterine NK cells in humans.

NK cells contribute to both beneficial and harmful immune responses, with their role being influenced by the immune response phase, the affected organ, and the NK cell subsets. The local microenvironment shapes the properties and functions of NK cells, influencing their activation, cytokine production, and cytotoxic potential. A unique subset of organ-resident NK cells has been characterized in the human decidua (dNK), the endometrium of the pregnant uterus that forms the maternal part of the placenta. Throughout the first and second trimesters of pregnancy, uterine (uNK) cells, accounting for around 70% of decidual leukocytes, remain steady, aligning with the period of trophoblast invasion. In the third trimester, however, their population declines significantly, although a small subset of these cells still persists (8, 9). uNK cells acquire several crucial functions: endometrial tissue remodeling, ensuring proper placentation through vascular remodeling through the production of proangiogenic factors, formation of a placenta in the uterus, and releasing chemokines that induce the migration of the extravillous cytotrophoblast, resulting in the remodeling of spiral arteries (10, 11). uNK cells originate from bone marrow (BM)-derived CD34+ cell precursors *in situ*, migrate from the peripheral blood through a CXCR3-dependent mechanism or local progenitor cells (8, 9, 12–14). The decidua is largely occupied by CD16^−^CD56^bright^ subset of NK cells which are characterized by low cytotoxicity, and high expression of cytokines, chemokines, angiogenic factors, activating receptors NKG2C and NKG2E (8, 15). Successful implantation is facilitated by dNK cells binding to non-classical MHC class I molecules HLA-E and HLA-G on trophoblasts which are the only fetal-derived cells in the maternal-fetal interface that express MHC class I antigens. Thus, uNK cells modulate immune tolerance and exert control over the degree of placental invasion. The characteristics of pNK and uNK cells were summarized in **Figure 1**.

The link between uNK cells and the vascular remodeling essential for healthy pregnancy development suggests that a malfunction in uNK cells could be a critical factor contributing to the onset of pregnancy complications such as pre-eclampsia, fetal growth restriction, or recurrent pregnancy loss. These complications are also a hallmark of antiphospholipid syndrome (APS), a systemic autoimmune condition driven by circulating antiphospholipid antibodies (aPL) directed against phospholipids or phospholipid-binding proteins such as β2-glycoprotein I (16, 17). The persistent presence of aPL gives rise to a wide range of clinical phenotypes, with thrombotic events and pregnancy complications being the most frequent, while numerous extra-criteria manifestations are also commonly observed (18). For a long time, hypercoagulation was considered the primary cause of pregnancy complications associated with APS. However, recent advancements in obstetric APS (OAPS) research have been highlighted the critical role of inflammation-mediated mechanisms in driving disease pathology, with contributions from both innate and adaptive immune components, including the emerging role of NK cells (19, 20).

The role of NK cells in autoimmunity is multifaceted, as they can either support protective immune responses or contribute to the development of autoimmune diseases, depending on the specific context and disease involved. Typically, NK cells exert a protective role by eliminating autoreactive Th17 and T follicular helper (Tfh) cell subsets, dampening excessive immune responses, thus preventing autoimmune reactions (21). Reduced cytotoxicity was documented in many autoimmune pathologies including systemic lupus erythematosus, multiple sclerosis, Sjogren’s syndrome (6). In contrast, overproduction of proinflammatory cytokines (IFN-γ and TNF-α) can amplify autoimmune responses and contribute to chronic inflammation. For example, T cell-dependent increase of IFN-γ production by NK cells may cause B cell proliferation, activation and support the production of autoantibodies (22). Another evidence is NK cell infiltration of near islets during the development of type I diabetes mellitus (T1DM) during prediabetic stage compared with late diabetes suggesting their contribution to the initiation of the autoimmune process (23). The contrasting functioning of NK cells within a single pathology and across different compartments of the organism was illustrated for rheumatoid arthritis (RA). In the joint synovium, NK cells contribute to the impairment of arthritis by producing GM-CSF, M-CSF and RANKL, thereby priming effector myeloid cells (24). While peripheral blood NK cells in secondary lymphoid tissues may exert a protective role by suppressing Th17 cell generation via IFN-γ production (25, 26). Likewise, the function of NK cells in APS varies across the compartments, particularly in regions most affected by disease pathogenesis, such as the peripheral blood and the placenta.

## 2 Peripheral blood NK cell in APS

Thrombotic and obstetrical complications are two common clinical hallmarks of APS. However, no studies have directly compared patients with ‘pure’ OAPS to those with ‘pure’ thrombotic APS in the context of NK cells, making it challenging to assess the potential role of NK cells in these distinct manifestations of APS. Generally, studies involving thrombotic APS patients have reported lower circulating NK cell counts, while higher NK cell counts are commonly observed in OAPS.

One of the few studies aiming to link NK cells with thrombotic risk in primary APS showed reduced absolute number and percentage of pNK cells, identified as CD16^+^CD56^+^ cells (27). Similarly, another comparative study demonstrated significantly reduced pNK cell numbers in APS patients with venous thromboembolism (VTE) compared to non-APS VTE patients and healthy controls (28). A very recent study showed that primary APS patients had a lower absolute number of pNK cells compared to controls, with an even more pronounced reduction observed in secondary APS patients (29). At the same time, these secondary APS patients exhibited lower absolute counts of other immune cells, including T and B cells This non-specific reduction in adaptive cell counts was thought to be related to immunosuppressive treatment (29). In contrast, an opposite pattern was reported in a cohort of APS patients with both thrombotic and obstetric manifestations, where a higher percentage of naïve B cells and activated T cell subsets, including CD4^+^DR^+^, CD8^+^DR^+^ cells, were observed (30). Further investigation confirmed that elevated levels of CD8^+^DR^+^ T cells represent an independent risk factor for APS-related thrombosis and neuropsychiatric manifestations (31). However, both the percentage and absolute count of pNK cells were again found to be lower in mentioned APS patients compared to controls (28).Contrary to coagulation processes, the importance of NK cells in pregnancy is broadly recognized. Consequently, the majority of studies on NK cells in APS have predominantly focused on obstetric cases. A significant challenge in systematizing the available knowledge is the variability in patient cohorts, including the enrolment of primary or secondary APS, timing of sampling in relation to the last pregnancy loss, and treatment protocols. Most of the published studies have reported quantitative differences in pNK cells in OAPS. The earliest report of pNK cell counts in OAPS dates back to 1995, when a significantly higher number of pNK cells was observed in aPL-positive women with recurrent spontaneous abortions (RSA) compared to aPL-negative RSA patients (32). The comparable values of pNK cell counts were found in APS patients without RSA (non-RSA) and healthy donors, similarly to other lymphocyte subpopulations, including CD4, CD8 and CD19 cells (33). While in OAPS patients with RSA, the absolute number and percentage of pNK cells were found to be significantly higher than in healthy donors, non-RSA APS patients and non-APS origin idiopathic RSA patients. Enrolled APS patients with RSA exhibited heterogeneity in terms of pNK cell percentages, and were further categorized into two groups: those with normal pNK cell levels (<15%) and those with elevated levels (>15%). Аs appeared, the majority of patients with pNK cell levels greater than 15% experienced abortions within the first 10 weeks of gestation, while patients with pNK cell levels below 15% had their last abortions beyond the 10th weeks of gestation. Notably, no correlation was observed in these patients between pNK cell count and the titres of any of aPL antibodies (33). Another evidence of a higher percentage of pNK cells was demonstrated in OAPS patients with abortions, preeclampsia or Hemolysis, Elevated Liver enzymes and Low Platelets (HELLP) syndrome when compared with uncomplicated aPL-positive pregnancies (34). In continuation of this study, after an initial pregnancy failure, patients were administered standard low molecular weight heparin (LMWH). During the follow-up, patients who experienced subsequent pregnancy loss were administered intravenous immunoglobulin (IVIg) during their next pregnancy, with treatment continuing until 32 weeks of gestation. This combined approach generally improved the rate of late pregnancy complications, presumably by influencing pNK cells as one of the underlying mechanisms. Although during follow-up the number of patients receiving IVIg treatment was insufficient to establish a strong statistical correlation between pNK cell count and pregnancy outcome, a tendency was still observed. Specifically, the degree of IVIg-induced reduction in pNK cell levels was positively correlated with a higher rate of live births, while elevated pNK cell levels were associated with pregnancy complications (34).

In addition to the study discussed above, the positive outcomes associated with the addition of IVIg to standard treatments for preventing APS-related pregnancy complications have often been reported (35, 36). Although the precise beneficial mechanism of IVIg has not been fully elucidated, it appears that IVIg inhibits aPL antibodies through anti-idiotypic activity, leading to a rapid but short-term improvement (35). The long-term effect of IVIg is presumably attributed to the inactivation of idiotype-bearing B cell clones, resulting in a subsequent decrease of their expansion (35). Additionally, IVIg was shown to inhibit NK activity *in vitro* in a dose-dependent manner (37). More recent studies have highlighted that in RSA patients, IVIg treatment during pregnancy may decrease pNK cell cytotoxicity and the expression of activating receptors such as KIR2DS1, KIR2DS4, and NKG2C, while increasing the expression of inhibitory receptors, namely KIR2DL1, KIR2DL2, KIR2DL3 and NKG2A (38). Alternatively, the IVIg therapeutic effect may be caused by NK and T cell interactions, as studies have shown that *ex vivo* IVIg-treated NK cells can induce expansion of CD4+Foxp3+ Treg cells *in vivo*, which may further suppress the progression of autoimmunity (39). Besides its suppressive effect on NK cell-mediated cytotoxicity, IVIg infusion also leads to a significant reduction in the number of pNK cells (40, 41). In patients with RSA, it has been reported that elevated numbers of circulating NK cells were significantly reduced by IVIg, leading to improved pregnancy outcomes (42). Despite the numerous attempts to adopt NK cells targeted treatment in women with reproductive failure (43), this approach remains highly controversial (44). The uncontrolled reduction in NK cell numbers, especially within placental tissue, may have a detrimental impact on pregnancy outcome. During the first trimester, decidua is overwhelmingly populated by NK, which played a crucial role in supporting pregnancy. Existing data indicate that IVIg treatment reduces NK cell counts in circulation, while no data available on its impact on uNK cells. It was demonstrated that IVIg exposure may induce caspase-3-dependent apoptotic death of peripheral blood cytotoxic CD56^dim^ cells, without affecting CD56^bright^ subset of NK cells (45). Considering that uterine-specific NK cells are predominantly CD56^bright^, selectivity in CD56^dim^ NK cells reduction poses IVIg as a potentially pregnancy-safe intervention; however, this assumption requires direct confirmation in the context of uNK cells. Less is known about the immunological effects of more widely used therapies such as low-dose aspirin, low-molecular-weight heparin (LMWH), and vitamin K antagonists (VKA). These agents represent the widely accepted therapeutic strategy for preventing pregnancy morbidity in patients with APS, and are also employed in other APS-related clinical contexts (46, 47). While their efficacy is primarily attributed to their antithrombotic properties, emerging evidence suggests potential immunomodulatory roles. Nevertheless, their direct effects on NK cell phenotype and function have not been systematically investigated, particularly within the context of APS-associated immune dysregulation and reproductive failure. In a recent study, aspirin was found to suppress VEGF expression through histone methylation in Epstein-Barr virus (EBV)-transformed NK cells (48). Although EBV+ NK cells differ from primary uNK cells, particularly in their receptor expression, cytokine responsiveness, and angiogenic capacity, the observed suppression of VEGF in EBV+ NK cells underscore the need for further research on the potential relevance of treatment used in APS to uterine immune regulation.

While the count of pNK cells is undoubtedly an informative parameter, the shaping of NK-mediated immune responses largely depends on their activation status, surface receptors repertoire, and the subpopulational distribution. Currently, only a few studies have been conducted on the detailed characterization of NK cells in APS. One of them focused on the subpopulation distribution and activation status of pNK cells in a cohort of APS patients with a history of pregnancy morbidity (49). Although the authors observed a tendency towards higher percentages of both the CD16+CD56dim and CD16−CD56bright pNK subsets within the total lymphocyte population in OAPS patients, the difference was not statistically significant (49). Subpopulation analysis demonstrated an increased proportion of NKG2A−NKG2D+ and a decreased proportion of NKG2A+NKG2D− subsets in both the CD16+CD56^dim^ and CD16−CD56^bright^ pNK cells in OAPS. The presence of a cytotoxic pNK cell in APS was further evidenced by an increased proportion of the CD27−CD11b+ subset within the CD16+CD56^dim^ pNK cells, known for its high cytolytic activity (49). While authors presented an expansion of pNK cells with phenotype corresponding to cytotoxic subsets in APS, functional assays were not conducted to provide mechanistic proof. In our recent *in vitro* study, the potential of aPL antibodies to induce pNK cell activation and cytotoxicity, through both phenotypic and functional assays, was demonstrated. Particularly, exposure of pNK cells, derived from healthy controls, to aPL IgG resulted in increased CD107a expression. Besides, a pro-activated phenotype was evidenced by increased levels of CD69, CD11b, and NKG2D across CD56^dim^CD16^bright^ and CD56^bright^CD16^dim^ NK cell subpopulations (50). The potential role of increased cytotoxic pNK cells in non-obstetric APS complications remains unclear. Recent findings suggest that IFN-γ-producing NK cells might play a contributory role in thrombus formation. Specifically, it has been shown that NK cells can promote venous thrombosis through IFN-γ-dependent neutrophil extracellular trap (NET) formation (51). The study proposed that IFN-γ secreted by NK cells induces NET formation by enhancing Ca^2+^ flux and reactive oxygen species (ROS) production in neutrophils.

## 3 Placental NK cells in APS

Due to ethical and technical challenges, evidence of uNK cell abnormalities in APS are extremely limited. It is important to acknowledge that numerous factors must be taken into account when aiming to investigate uNK cells. The choice of tissue, such as endometrium or decidua, the timing of sample collection and proper selection of control group will ultimately affect the final result. Endometrial NK cell number progressively increases throughout the menstrual cycle to prepare the endometrium for implantation (52). NK cells in the non-pregnant endometrium have different phenotype and NKR repertoire compared to those in early-pregnancy decidua, and characterized by higher expression of KIRs and lower levels of activating receptors such as NKG2D, NKp30, and NKp46 (53). During the established pregnancy, hormonal fluctuations may represent one of the mechanisms regulating the dynamics of NK cell composition in the uterus. Specifically, estrogens regulate various functions of NK cells, including their recruitment, expansion, secretory and pro-angiogenic activities (43, 54). At levels corresponding to pregnancy, estrogens suppress NK cell cytotoxicity and downregulate the expression of NK activating receptors (55, 56). In patients at risk of miscarriage, estrogen levels are significantly lower and decline even further after a miscarriage (57), diminishing its suppressive effect on NK cells. Therefore, the activation status of uNK cells in women selected as the control group will greatly depend on their pregnancy status and the week of gestation. It may vary significantly between individuals undergoing elective abortion and those experiencing spontaneous miscarriages.

The cyclic expansion of uNK cells during the late secretory phase is essential for stromal cell decidualization and fetal trophoblast invasion, processes that are crucial for successful implantation (58). There is evidence indicating that positivity for aPL does not impact the number of endometrial NK cells in non-pregnant patients with the history of RSA or recurrent implantation failure (RIF) (59). These findings seem reasonable, as early pregnancy loss is a more common complication for APS patients than implantation failure. Accordingly, there is a possibility that pregnancy-threatening processes, including NK-mediated deregulation, potentially develop after implantation. Among the rare reports on uNK cell composition in APS decidua, one analyzed decidua specimens collected by curettage from women with refractory aPL-mediated RSA following pregnancy loss before 14 weeks of gestation, compared to a control group of patients undergoing elective termination of viable pregnancies within the same gestational period. The authors reported an overexpression of CD16+CD56^dim^ uNK cells in APS, as determined by immunohistochemical analysis of decidual specimens (60). Nonetheless, in our view, the study has notable limitations. First and foremost, immunohistochemistry is not the most reliable method for detailed immune cell phenotyping, as it lacks the precision and resolution required to characterize immune cell subsets accurately (61). Second, the study analyzed the number of patients stained positive for one of uNK subsets, namely CD56^dim^CD16+ or CD56^bright^CD16−, rather than examining the proportional distribution of these subsets within the two investigated groups. This approach limits the ability to draw comprehensive conclusions about the relative changes in uNK subset composition between the groups.

Emerging studies suggest that the importance of uNK cell count for pregnancy outcome may be overestimated, with inappropriate activation playing a more decisive role (62). A recent systematic review and meta-analysis suggested that there is no significant correlation between pNK and uNK cell levels in the endometrial or decidual tissues of women with RSA or RIF (63). In our recent study, uNK cells from mid-pregnancy mice placentas in pre-abortion settings were collected to investigate the ongoing pathological mechanisms that may contribute to APS-associated pregnancy morbidity. Transcriptional profiling of uNK cells revealed a high number of differentially expressed genes, with the majority being down-regulated in APS (20). Cytokine-cytokine interactions, lysosome, protein processing in endoplasmic reticulum and PI3K-AKT signaling, were among the top enriched GO terms of down-regulated uNK genes. Analysis of upregulated genes did not reveal any terms related to cell activation. We proposed that the presence of anti-β2GPI antibodies reshape the uNK cells into a partially dysfunctional state, which eventually contributed to the pathological alterations observed in placental tissue. Examination of placental vascularization revealed another potential indicator of uNK dysfunction. Thinning of vessel walls and reduced levels of VEGF-A could be associated with disrupted pro-angiogenic and secretory properties of uNK cells in APS (20). It remained uncertain whether the observed transcriptome profile is driven by exhaustion or senescence mechanisms. Typically, both these processes are associated with a reduction of effector functions of the cells (64). In the placenta, diminished NK cell functionality could result from prolonged exposure to aPL antibodies within the placental microenvironment. This phenomenon is similar to what has been observed in chronic stimulation conditions, such as in tumors (64, 65). NK cells can undergo metabolic exhaustion from prolonged activation, leading to mitochondrial dysfunction, disrupted glucose metabolism, and increased oxidative stress. These factors may work together to diminish the energy of NK cells to uphold their effector functions.

Clinical evaluation of pregnancy outcomes in the APS animal model, combined with morphological and transcriptomic analyses of placentas, identified multiple indicators suggestive of preeclampsia (20). The number and status of uNK cells, whether activated or inhibited, appear to be critical for the success of pregnancies. Any imbalance in uNK cells may compromise their ability to perform their normal functions, potentially leading to pregnancy complications such as preeclampsia. Reproductive failure continues to be attributed to exaggerated NK cell responses, leading to ongoing suggestions to score NK activity and percentage as predictors of pregnancy outcomes and to develop NK-targeted therapies (9, 66). Consistent with this hypothesis, some studies have reported NK cell overactivation in preeclampsia. A shift to cytotoxic behavior, characterized by elevated intracellular IFN-γ, perforin and granzyme B, has been observed both in dNK and pNK cells among preeclamptic patients (67). Another study linked the enhanced cytotoxic capacity of pNK cells in preeclampsia to increased expression of the activating receptor NKG2D (68, 69).

Recent advancements in the field suggest that the maternal immune system remains active and functional during pregnancy, with immune cells at the implantation site, particularly NK cells, not requiring suppression to achieve immune tolerance toward the fetus (70–72). While excessive activation of NK cells may potentially contribute to the development of preeclampsia by inducing trophoblast death, insufficient activation of uNK cells can be equally detrimental, leading to inadequate spiral artery remodeling, reduced secretion of angiogenic factors, and insufficient trophoblast invasion. In line with our findings from the mouse model, accumulating evidence of diminished activation of uNK cells were documented in human preeclamptic placentae. A recent study revealed that a significant portion of uNK cells within the preeclamptic decidua are in an immature state (73). Authors proposed that hypoactive dNK subpopulation, identified as CD56+CD3-NKp46+/−NKp30+NKG2D+IFN-γ+, is particularly linked to preeclampsia (73). Investigation of pregnancies at higher risk of preeclampsia revealed that uNK cells exhibited weaker control over trophoblast migration and invasion (74). This observation could be linked to the reduced secretory capacity of dNK cells, potentially limiting spiral artery transformation and contributing to hypertension in pregnant women and eventually to preeclampsia (74).

Current knowledge regarding uNK cells in APS-complicated pregnancies remains highly limited. Our recent study shed a light on diminished uNK cell activity in the APS mouse model. This effect cannot be solely attributed to aPL exposure, as aPL antibodies are known to activate innate immune cells, including NK cells, independent of pregnancy. We propose that the intricate cooperation among various placenta-forming cells and their soluble mediators within the aPL milieu plays a pivotal role in modulating uNK cell states and remodeling placental architecture. Particularly, among non-immune cells, trophoblast and endothelial cells likely have significant input, as they are primarily targeted and can be activated by aPL antibodies. Decidual Tregs represent another critical regulator of uNK cell function that warrants detailed evaluation, as Treg-mediated suppression of uNK cells is one of contributing mechanisms of preeclampsia. Hence, further investigation of human APS pregnancies, with a focus on interactions at both the humoral and cellular levels, will be invaluable for validating existing findings and elucidating the underlying mechanisms.

## 4 Conclusion

It is well-established that NK cells exhibit dual and context-dependent roles in both peripheral blood and placenta. This duality becomes increasingly intricate in APS due to the complex interplay between the cells, decidual microenvironment and aPL. There are valid reasons for caution and skepticism in studying the role of uNK cells using peripheral blood NK cells, especially in the context of conditions like APS. Extrapolating findings from pNK studies to uNK contexts risks overlooking critical uNK-specific functions. Although information on uNK cells in APS remains limited, evidence suggests that uNK cells in APS are dysfunctional. Undoubtedly uNK cells are key players in the pathologies associated with the placenta in APS. The altered activity of NK cells, potentially driven by aPL, and the decidual microenvironment can affect placental development, vascular remodeling, and promote adverse pregnancy outcomes, including recurrent pregnancy loss, fetal growth restriction, and preeclampsia, which are commonly observed in APS. Recognizing their crucial role provides an opportunity for future research to better elucidate the mechanisms underlying their dysfunction and to explore therapeutic strategies targeting uNK cells, which could mitigate placental complications in APS.
